# The effect of amylase, chromium propionate and their combination supplementation on growth performance, carcass traits, serum parameters, antioxidant capacity and intestinal health in yellow feathered broilers

**DOI:** 10.1016/j.psj.2025.105229

**Published:** 2025-04-29

**Authors:** Kun Chen, Junran Lv, Zheng Luo, Zhuang Liu, Mingzhu Cen, Benkuan Li, Jiancun Ou, Huihua Zhang

**Affiliations:** a Department of Food and Drug Technology, Shandong Vocational Animal Science and Veterinary College, Weifang 261061, China; b School of Animal Science and Technology, Foshan University, Foshan 528225, China; c Kemin (China) Technologies Co., Ltd., Zhuhai, China

**Keywords:** Yellow-feathered broiler, Amylase, Chromium propionate, Growth performance, Healthy parameters

## Abstract

To better understand the growth promotion mechanism of amylase and chromium propionate (Cr Prop) and assess their potential synergistic effects, a total of 640 one-day-old male yellow-feathered broilers were randomly allocated to four dietary treatments with eight replicates. The birds were fed either a basal diet or the basal diet supplemented with amylase, Cr Prop or a combination of both. The results showed that during the grower, finisher and overall phases, average daily feed intake, final body weight, and feed conversion ratio were significantly improved (*P < 0.05*) in the amylase and Cr Prop treatment groups. However, no significant differences (*P > 0.05*) were observed in carcass traits. Amylase supplementation alone significantly reduced breast muscle drip loss (*P < 0.05*). Amylase supplementation significantly increased (*P < 0.05*) the concentration of glutathione peroxidase in breast muscle and plasma, as well as catalase in breast muscle, while it decreased (*P < 0.05*) catalase in the liver and malondialdehyde in breast muscle. Supplementation with Cr Prop significantly elevated (*P < 0.0*5) the concentration of glutathione peroxidase in the liver and plasma, as well as the concentration of total superoxide dismutase in the liver, while it reduced (*P < 0.05*). the concentration of malondialdehyde in breast muscle. Supplementation with either amylase or Cr Prop significantly increased (*P < 0.05*) the levels of blood high-density lipoprotein cholesterol, immunoglobulin A, immunoglobulin G, and total protein, while significantly reducing (*P < 0.05*) triglyceride levels. Amylase supplementation alone notably enhanced villus height in both the jejunum and ileum. Furthermore, amylase or Cr Prop significantly upregulated (*P < 0.05*) the mRNA expression of Occludin and Claudin-1 in the Jejunum. The expression of Zona Occluden-1 was elevated by Cr Prop. However, the expression of Mucin 2 and Zona Occluden-2 remained unaffected. While amylase or Cr Prop supplementation alone could improve the performance and several healthy parameters in yellow-feathered broilers, no synergistic effect was observed.

## Introduction

In China, yellow feathered-broilers are the second-largest source of poultry meat, with annual consumption exceeding four billion birds (Li et al., 2019). These broilers are particularly popular in southern China due to the high quality and flavor of their meat (Gou et al., 2016; Wang et al., 2021). However, yellow-feathered broilers are characterized by slow growth performance, including longer growing periods, a higher feed conversion ratio (FCR) ranging from 1.8 to 2.6 (Tang et al., 2021) and increased abdominal fat deposition compared to white-feathered broilers (Weng et al., 2022) Improving feed efficiency and carcass quality is therefore critical for the sustainable development of the yellow-feathered broiler industry.

Dietary energy and protein are essential for optimizing growth performance in poultry (Liu and Selle, 2015). Starch, as the primary energy source in broiler diets, has been shown to significantly influence both growth performance and carcass traits in broiler (Khoddami et al., 2018). The efficiency of starch digestion depends on several factors, including the bird’s growth phase (Aderibigbe et al., 2020), dietary composition and the chemical structure of the starch (Tester et al., 2004). While endogenous amylase plays a key role in starch digestion, a proportion of dietary starch remains resistant to digestion in the small intestine (Vasanthan and Bhatty, 1996). Supplementation with exogenous amylase has been reported to improve the digestion of resistant starch in corn-soybean meal diets (Aderibigbe et al., 2020; Bassi et al., 2023), although some studies found no significant effects on growth performance, carcass characteristics, or organ weight (Radhi et al., 2023). The dosage of exogenous α-(1,4)-amylase is important, as more than 600 U/kg α-(1,4)-amylase significantly elevated the glucose concentration in serum, which in turn limits the growth performance of broiler chickens (Zhou et al., 2021). If the body tissue of birds can speed up the utilization of glucose, then a higher dosage of α-(1,4)-amylase may continue to increase their performance. However, no such research has been found specifically in yellow-feathered broilers.

Chromium (Cr), an essential trace mineral, played a vital role in carbohydrate and lipid metabolism, as well as for protein and nuclei acid synthesis (Mertz, 1993). While Cr can be provided in both organic and inorganic form, studies have demonstrated that organic forms, such as chromium propionate, have superior bioavailability (Pierce, 2009). Previous studies have shown that Cr Prop supplementation can affect plasma lipid concentrations (Anderson, 1995), growth performance (Rosebrough and Steele, 1981), carcass characteristics (Ohh and Lee, 2005), antioxidant properties, physiological functions, including cell preservation and immune response (Dalio et al., 2018), and plasma glucose levels (Mátis et al., 2014). It was reported that 200 – 1200 ppb of Cr Prop can reduce serum glucose to a level without significant difference (Muhammad Arif et al., 2019). The main mechanism by which chromium helps glucose utilization is through insulin pathway (Brooks et al., 2016 and Spears et al., 2024). As both amylase and chromium play important roles in optimizing the utilization of sugar in broilers, and no studies have directly compared the effects of amylase and Cr Prop, not to mention their potential synergistic effects. Therefore, the aim of this study was to investigate their individual and combined effects on growth performance, carcass traits, antioxidant capacity, and intestinal health in yellow-feathered broilers.

## Materials and methods

All animal care and experimental procedures in this study were approved by the Animal Care and Use Committee of Foshan University, China.

### Experimental design and diets

A total of 640 one-day-old male yellow-feathered broilers were individually weighed and randomly assigned to one of four treatment groups. Each group consisted of 8 replicates with 20 birds per replicate. The experiment comprised four dietary treatments: (A) basal diet (BAS), (B) BAS + 150 mg/kg amylase (Amy), (C) BAS + 200 mg/kg chromium propionate (Cr), and (D) BAS + 150 mg/kg amylase + 200 mg/kg chromium propionate (Amy-Cr). The basal diet was the control group. The basal diet (corn-soybean meal based) was formulated to meet or exceed the nutrient requirements of yellow-feathered broiler chickens (NY/T3645-2020). The feed ingredients and dietary nutrient compositions are listed in Table 1. Amylase (4000 U/g) and chromium propionate (0.1% Cr Prop) were provided by Kemin (China) Technologies Co., Ltd., (Zhuhai, China).

**Table 1:** Feed composition offered to broilers during experiment.

Ingredients (100%)		1-21d	22-42 d	43-56 d
Corn		44.50	39.50	42.20
Soy bean meal		33.50	33.00	27.70
Extruded soybean		5.00	0.00	0.00
Sorghum		10.00	20.00	20.00
Lard		1.50	3.50	6.40
Imported fish meal	1.30		0.00	0.00
Dicalcium phosphate	1.30		1.30	1.20
Limestone	1.40		1.40	1.00
L-lysine hydrochloric acid	0.25		0.19	0.24
DL-methionine	0.23		0.21	0.19
L-threonine	0.05		0.06	0.05
Premix compound^1^	0.71		0.57	0.75
Sodium chloride	0.12		0.15	0.15
Choline chloride	0.12		0.10	0.10
Antioxidant	0.01		0.01	0.01
Phytase	0.01		0.01	0.01
Calculated nutrient composition				\
Crude protein	21.45		19.09	17.11
Metabolic Energy kcal/kg	2877.00		2956.05	3173.48
Crude fat	4.87		6.02	8.85
Calcium	1.00		0.94	0.75
Phosphorus	0.64		0.60	0.56
Lysine	1.39		1.16	1.06
Methionine	0.56		0.49	0.45
Threonine	0.88		0.78	0.69

1 Premix provided to per kilogram of the diet: vitamin A 12000 IU; vitamin D_3_ 3000 IU; vitamin E 10 IU; vitamin K_3_ 2 mg; vitamin B_1_ 1 mg; vitamin B_2_ 3 mg; vitamin B_6_ 2 mg; vitamin B_12_ 0.01 mg; niacin 20 mg; pantothenic acid 4 mg; folic acid 0.54mg; biotin 0.05 mg; Fe 100 mg; Cu 20 mg; Mn 100 mg; Zn 80 mg; I 3 mg; Se 0.5 mg

### Housing and management

The trial was conducted at the scientific research broiler farm of Foshan University, Foshan, China. The total duration of the trial was 56 days, divided into three phases: starter (d 1-21), grower (d 22-42), and finisher (d 43-56). Broilers were housed in 2-layer stainless steel cages (300 cm × 80 cm × 60 cm) with 6 nipple drinkers and 2 feed troughs per cage. During the first 3 days, the house temperature was kept at 33 °C ∼ 34°C, decreasing by 3°C per week until a final temperature of 24 °C was reached. Lighting was continuous (24 h), and relative humidity was kept at 60 ∼ 70%. Birds had free access to water and feed throughout the study.

### Growth performance

Initial body weight (IBW) was recorded on day 1 for all birds. On days 1, 22, 43, and 57, birds were weighed by cage after a 12-hour fast. Feed consumption was recorded at each weighing to calculate average daily gain (ADG), average daily feed intake (ADFI) and feed conversion ratio. Birds with severe leg deformities or significantly below the average body weight of their treatment group were excluded from the trial.

### Carcass traits measurements

At d 56, one bird per cage, closest to the average body weight of the treatment group, was selected for slaughter. A total of 8 broilers per treatment group, with similar live weights, were seuthanized by cervical dislocation, followed by bleeding for 5 minutes. Feathers removal was processed after submerging in hot water of 60 °C for 2 min. Carcass weight was recorded after bleeding and feather removal. Trachea, esophagus, crop, intestines, spleen, pancreas, gut, bursa of Fabricius, head, feet, liver, gizzard, and abdominal fat were removed by skilled personnel. Weights were determined for skinned breast meat (2 sides), leg meat (2 sides; thigh + calf) without bones. Semi-eviscerated yield and eviscerated yield were calculated as percentage of live body weight, while breast and thigh meat yields were expressed as percentages of eviscerated weight. Abdominal fat yield was calculated as a percentage of carcass weight.

### Meat quality evaluation

On day 56, left breast and the left leg muscles from 8 birds per treatment group were collected and used for meat quality (pH, color, drip loss, and shear force) measurements.

*pH value*. The pH of breast meat was measured at 45 minutes and 24 hours post-slaughter using a probe (Testo 205, AG, Lenzkirch, Germany). Three measurements were taken at random muscle locations, and the average value was recorded.

*Meat color*. Meat color was assessed immediately after muscle fat was removed, using a Minolta reflectance colorimeter (CR-300, Konica Minolta Sensing, Osaka, Japan). The brightness (L*) was measured by the chroma meter with a 50 mm- reading head. Three random readings were performed on each sample.

*Drip loss*. a 3 cm-thick cube of breast meat was weighed (weight 1), sealed in a plastic bag and stored for 24 h at 4°C. After wiping the surface moisture, the samples were weighed (weight 2). Drip loss was calculated as the percentage of weight lost.

*Shear force*. After cooking, shear force was measured using a C-LM3 digital tenderness meter (Bulader Co., Ltd, Beijing, China). Each sample was cut transversely and tested at a speed of 1 mm/s. The maximum shear force was recorded, and all determinations were conducted in triplicate.

### Sample collection and plasma biochemical measurements

On day 56, after a 12-hour fast, 16 birds per treatment group were selected based on average body weight. Blood samples (5 mL) were collected from wing vein, and serum was obtained by centrifuging blood sample at 3000 × *g*, 4°C for 15 min and stored at -20°C. Serum samples were processed for analysis of total cholesterol (TCH; Catalog No. A111-1), triglyceride (TG; Catalog No. A110-1), high-density lipoprotein cholesterol (HDLC; Catalog No. A112-2), low density lipoprotein cholesterol (LDLC; Catalog No. A113-2), immunoglobulin A (IgA; Catalog No. E027-1), immunoglobulin M (IgM; Catalog No.H109-1), immunoglobulin G (IgG; Catalog No. E026-1), corticotropin releasing hormone (CRH; Catalog No. H288-1), creatine kinase (CK; Catalog No. A032-1), corticosterone (CORT; Catalog No. H205-1), albumin (ALB; Catalog No. A028-2), total protein (TP; Catalog No. A045-2), glutamic-pyruvic transaminase (ALT; Catalog No. 009-3), glutamic oxalacetic transaminase (AST; Catalog No. 010-3), glucose (Glu; Catalog No. F006-1), lactate dehydrogenase (LDH; Catalog No. A020-2). The broiler was euthanized after blood sampling. The right lobe liver tissues were sampled, quickly frozen in liquid nitrogen, and kept at -80°C. Commercial kits for these analyses were purchased from Nanjing Jiancheng Bioengineering Institute (Nanjing, China).

### Antioxidant parameters

On day 56, liver tissues and serum samples from 8 birds per treatment group were analyzed for antioxidant activity. Parameters measured included total superoxide dismutase (T-SOD; Catalog No. A001-3), glutathione peroxidase (GSH-Px; Catalog No. A005-1), malondialdehyde (MDA; Catalog No. A003-1), total antioxidant capacity (T-AOC; Catalog No. A015-2), and catalase (CAT; Catalog No. A007-1). Commercial kits for these assays were purchased from Nanjing Jiancheng Bioengineering Institute. (Nanjing, China).

### Intestinal morphometry

On day 56, segments of duodenum, jejunum, and ileum from 8 birds per treatment group were collected for histomorphometric analysis. Tissues were fixed in formalin, sectioned at 1 cm thickness, and stained with hematoxylin and eosin. Villi height (VH), crypt depth (CD), and the villus-to-crypt ratio (V/C) were measured using a Leica DM3000 microscope (Leica Microsystems, Germany). Additionally, 2 cm sections of jejunum were washed in ice-cold saline, stored at -80°C, and used for further molecular analysis.

### Quantitative real-time PCR

Total RNA was extracted from jejunal tissues of 8 birds per treatment group using TRIzol reagent (Vazyme, Nanjing, China) and reverse transcribed to cDNA using the Hiscript III RT SuperMix kit (Vazyme, Nanjing, China). RNA concentration and purity (A 260/280 ratio) were checked using a spectrophotometer (NanoDrop 2000, Thermo Fisher Scientific, Inc., Wilmington, DE, USA). Primers listed in Table 2 were designed according to NCBI sequences. The PCR reaction was conducted in Applied Biosystems QuantStudio5 (Thermo Fisher, USA) with the procedures of 95°C for 30 s, 40 cycles at 95°C for 10 s, and 60°C for 30 s, and the melting curve period (95°C for 15 s, 60°C for 60 s, and 95°C for 15 s). The 20 μL reaction system is composed of 1 μL cDNA, 0.4 μL forward and reverse primers, 8.2 μL ddH2O, and 10 μL SYBR qPCR Green Master Mix (Vazyme, Nanjing, China). Each sample was triplicated in the assay. Gene expression data were analyzed using the 2‐ΔΔCt method (Livak and Schmittgen, 2001) with β-actin as the reference gene.

**Table 2:** Primer sequences used in quantitative real-time PCR.

Genes	GenBank accession NO.	Primer sequences (5’—3’)	Product length/bp	Annealing temperature/°C
β-actin	NM_205518.2	F: CATTGTCCACCGCAAATGCT	109	57.2
		R: AAGCCATGCCAATCTCGTCT		
Occludin	XM_046904539.1	F: TGCTTTTGCCCAAGCAGGAA	559	55.8
		R: TGTGGGAGAGGCACCAGTTG		
Claudin-1	NM_001013611.2	F: GGTATGGCAACAGAGTGGCT	91	57
		R: CAGCCAATGAAGAGGGCTGA		
MUC-2	XM_040673059.2	F: TTCATGATGCCTGCTCTTGTG	93	58
		R: CCTGAGCCTTGGTACATTCTTGT		
ZO-1	XM_046925214.1	F: CTTCAGGTGTTTCTCTTCCTCCTC	131	56.6
		R: CTGTGGTTTCATGGCTGGATC		
ZO-2	XM_015280244.4	F: GCAGAGACAACCCCCACTTT	148	56.2
		R: CGTTAACCATGACCACCCGA		

### Statistical analysis

Data were analyzed by one-way ANOVA using SPSS19.0 (SPSS Inc., Chicago, IL, USA). Differences between treatment groups were determined using Tukey’s multiple comparison test. Statistical significance was set at *P < 0.05*.

## Results

### Growth performance

The growth performance results were summarized in Table 3. During the starter stage, the ADFI in Group Amy-Cr was significantly lower than Group BAS (*P = 0.0032*), Amy group (*P = 0.0377*), and Amy-Cr group (*P = 0.0104*). Meanwhile the FCR of Group Amy- Cr showed significant improvement compared to all other groups (*P* values: BAS vs. Amy-Cr = 0.0035, Amy vs. Amy-Cr = 0.0165, and Cr vs. Amy-Cr = 0.0066). In the grower stage, both FBW (*P = 0.0023*) and ADG (*P* = *0.0252*) were significantly improved in Group Cr compared with Group BAS. During the finisher stage, the FBW (*P = 0.0369*), ADG (*P = 0.0269*), and FCR (*P = 0.0372*) were significantly improved in Group Cr compared with Group BAS. The ADG (*P = 0.0342*) and FCR (*P = 0.0109*) showed significant improvement in Group Amy-Cr compared with Group BAS. Over the entire experiment period (day 1-56), FBW (*P = 0.0359*), ADG (*P = 0.0208*) and ADFI (*P = 0.0009*) were significantly improved in Group Cr compared to Group BAS. The ADFI of Group Amy (*P = 0.0064*) was higher than that of Group BAS, while there was no difference compared to other groups. However, no significant synergistic effect was observed from the combined supplementation of amylase and Cr Prop.

**Table 3:** Effects of dietary amylase, Cr Prop, and their combination on growth performance in yellow-feathered broilers.

Items	BAS	Amy	Cr	Amy-Cr	*P-*value					
					BAS vs. Amy	BAS vs. Cr	BAS vs. Amy-Cr	Amy vs. Cr	Amy vs. Amy-Cr	Cr vs. Amy-Cr
Initial Body Weight (g)	34.26	34.10	34.00	34.00	0.9223	0.7409	0.7409	0.979	0.979	>0.9999
21d FBW (g)	411.60	405.80	409.20	395.20	0.8536	0.9871	0.1409	0.965	0.4776	0.2472
1-21d ADG (g)	18.86	18.58	18.74	18.04	0.8524	0.9846	0.1261	0.9687	0.4443	0.2317
1-21d ADFI (g)	29.64	29.04	29.36	27.46	0.685	0.9533	0.0032	0.9326	0.0377	0.0104
1-21d FCR	1.57	1.56	1.57	1.52	0.8989	0.9924	0.0035	0.9752	0.0165	0.0066
42d FBW (g)	1219.82	1245.18	1258.70	1230.38	0.0572	0.0023	0.6708	0.481	0.4039	0.0296
22-42d ADG (g/d)	38.18	39.52	39.85	39.28	0.0904	0.0252	0.1993	0.9228	0.9712	0.7154
22-42d ADFI (g/d)	81.13	81.00	83.02	82.78	0.9992	0.2949	0.4054	0.2415	0.3397	0.9958
22-42 d FCR	2.12	2.07	2.11	2.13	0.3375	0.996	0.9922	0.4547	0.22	0.9579
56d FBW (g/d)	1891.12	1943.78	1951.78	1939.90	0.081	0.0369	0.116	0.9795	0.9975	0.9378
43-56d ADG (g/d)	45.42	49.25	50.00	49.83	0.0762	0.0269	0.0342	0.9566	0.9787	0.9995
43-56d ADFI (g/d)	141.47	144.73	142.58	142.63	0.0542	0.7821	0.7591	0.2951	0.3143	>0.9999
43-56d FCR	3.17	3.01	2.88	2.82	0.4164	0.0372	0.0109	0.5311	0.244	0.9403
1-56d FBW (g/d)	1857.08	1909.75	1917.75	1905.87	0.0797	0.0359	0.1132	0.9788	0.9977	0.9378
1-56d ADG (g/d)	33.16	34.10	34.25	34.03	0.0535	0.0208	0.0633	0.9691	0.9998	0.9503
1-56d ADFI (g/d)	77.02	78.83	79.28	78.33	0.0064	0.0009	0.0581	0.8136	0.7374	0.2566
1-56d FCR	2.32	2.31	2.32	2.30	0.9048	0.9999	0.8155	0.9281	0.9969	0.8482

BAS: basal diet; Amy: BAS + amylase; Cr: BAS + chromium propionate; Amy-Cr: BAS + amylase + chromium propionate. Differences between treatment groups were determined using Tukey’s multiple comparison test. Statistical significance was set at *P < 0.05*. There were 8 replications per treatment group, and 20 birds per replication. On days 1, 22, 43, and 57, all birds were weighed by cage after a 12-hour fast.

### Carcass traits

The detailed carcass characteristics of all treatment groups were presented in Table 4. No significant differences (*P > 0.05*) were observed in carcass weight, eviscerated yield, semi-eviscerated yield, abdominal fat yield, or breast and thigh muscle yield among all treatment groups.

**Table 4:** Carcass parameters respond to dietary amylase, Cr Prop, and their combination on d 56.

Items	BAS	Amy	Cr	Amy-Cr	*P-*value					
					BAS vs. Amy	BAS vs. Cr	BAS vs. Amy-Cr	Amy vs. Cr	Amy vs. Amy-Cr	Cr vs. Amy-Cr
Dressing percentage (%)	85.15	87.58	88.40	88.29	0.5101	0.2903	0.3158	0.9606	0.9741	0.9999
Semieviscerated yield (g)	78.93	81.69	82.79	81.95	0.4099	0.1776	0.3393	0.9105	0.9984	0.9575
Eviscerated yield (g)	63.19	65.11	67.26	64.81	0.5694	0.0856	0.6895	0.4861	0.9962	0.3815
Breast yield (%)	14.91	14.53	14.24	13.22	0.9791	0.9016	0.3756	0.9904	0.5708	0.7345
Thigh yield (%)	21.54	20.73	21.44	21.07	0.6966	0.999	0.9166	0.77	0.9644	0.9562
Abdominal fat (%)	3.74	2.70	3.80	4.29	0.3025	0.9995	0.7596	0.2632	0.0785	0.8153

BAS: basal diet; Amy: BAS + amylase; Cr: BAS + chromium propionate; Amy-Cr: BAS + amylase + chromium propionate. Differences between treatment groups were determined using Tukey’s multiple comparison test. Statistical significance was set at *P < 0.05*. On day 56, 8 birds per treatment group were selected for slaughter.

### Meat quality indices

Meat quality results were presented in Table 5. Drip loss was significantly reduced in Group Amy compared to Group BAS (*P = 0.0218*) and Group Cr (*P = 0.0043*), whereas there was no difference compared to Group Amy-Cr (*P = 0.7373*). The drip loss of Group Amy-Cr was lower than Group Cr (*P = 0.0158*). No significant differences were observed in other meat quality parameters, including pH, color, or shear force, among the treatment groups.

**Table 5:** Meat quality responds to dietary amylase, Cr Prop, and their combination at d 56.

Items	BAS	Amy	Cr	Amy-Cr	*P-*value					
					BAS vs. Amy	BAS vs. Cr	BAS vs. Amy-Cr	Amy vs. Cr	Amy vs. Amy-Cr	Cr vs. Amy-Cr
pH_45min_	6.52	6.50	6.38	6.26	0.9994	0.8524	0.4869	0.8995	0.5479	0.8995
pH_24h_	6.59	6.47	6.51	6.16	0.9501	0.9847	0.2791	0.9977	0.5168	0.4248
Drip loss 24h (%)	1.21	0.62	1.40	0.78	0.0218	0.606	0.092	0.0043	0.7373	0.0158
Meat color L*	84.07	78.81	80.96	80.41	0.4255	0.7792	0.6889	0.91	0.9591	0.9982
Shear force (kg·f)	29.34	29.22	24.87	24.09	>0.9999	0.858	0.7925	0.8669	0.8029	0.999

BAS: basal diet; Amy: BAS + amylase; Cr: BAS + chromium propionate; Amy-Cr: BAS + amylase + chromium propionate. Differences between treatment groups were determined using Tukey’s multiple comparison test. Statistical significance was set at *P < 0.05*. On day 56, 8 birds per treatment group were collected and used for meat quality (pH, color, drip loss, and shear force) measurements.

### Antioxidant parameters

Antioxidant indices were presented in Table 6. In the liver, the concentration of GSH-Px in Group Cr was higher than Group BAS (*P = 0.0006*) and Group Amy (*P = 0.0056*). Similarly, the concentration of GSH-Px in Group Amy-Cr was increased compared with Group BAS (*P = 0.0012*) and Group Amy (*P = 0.0121*). There was no difference between Group Cr and Group Amy-Cr. The concentration of CAT in Group Amy was lower than Group BAS (*P= 0.0065*) and Group Amy-Cr (*P = 0.0037*). The concentration of T-SOD in Group Cr was significantly increased compared with all other groups (*P < 0.0001*). In breast muscle, the concentration of GSH-Px in Group Amy was higher than that in Group BAS (*P = 0.0007*) and Group Cr (*P = 0.0077*). The concentration of GSH-Px was significantly increased in Group Amy-Cr compared with Group BAS (*P < 0.0001*) and Group Cr (*P = 0.0004*). The CAT level in Group Amy was higher than that in Group BAS (*P = 0.0399*). The MDA concentrations of Group Amy and Group Cr were significantly lower than those in Group BAS and Amy-Cr (*P < 0.0001*). In plasma, compared with Group BAS, the GSH-Px levels were significantly higher in Group Amy (*P = 0.006*), Group Cr (*P = 0.0088*), and Group Amy-Cr (*P = 0.0027*). The T-AOC levels were elevated in Group Amy (*P = 0.036*) and Group Cr (*P = 0.028*) compared with Group Amy-Cr.

**Table 6:** Effects of dietary amylase, Cr Prop, and their combination on antioxidation in yellow-feathered broilers.

Items	BAS	Amy	Cr	Amy-Cr	*P-*value					
					BAS vs. Amy	BAS vs. Cr	BAS vs. Amy-Cr	Amy vs. Cr	Amy vs. Amy-Cr	Cr vs. Amy-Cr
Liver										
GSH-Px (U/mgprot)	41.75	51.80	76.72	73.61	0.2795	0.0006	0.0012	0.0056	0.0121	0.9274
CAT (U/mg)	77.82	62.34	71.54	79.30	0.0065	0.293	0.9671	0.0868	0.0037	0.1604
T-SOD (U/mL)	36.56	36.87	41.13	37.00	0.5872	<0.0001	0.3153	<0.0001	0.9413	<0.0001
T-AOC (mM)	10.73	12.36	12.11	11.64	0.558	0.672	0.8736	0.9967	0.9285	0.9772
MDA (nmol/mL)	1.26	0.97	1.07	1.12	0.4628	0.7606	0.8923	0.9449	0.8397	0.9916
Breast										
GSH-Px (U/mgprot)	15.79	22.8	17.96	25.58	0.0007	0.2441	<0.0001	0.0077	0.1106	0.0004
CAT (U/mg)	0.10	0.27	0.19	0.23	0.0399	0.3479	0.1334	0.4385	0.8262	0.8843
T-SOD (U/mL)	11.21	12.65	10.87	11.04	0.1673	0.9397	0.991	0.0759	0.1121	0.992
T-AOC (mM)	0.16	0.16	0.15	0.17	>0.9999	0.9755	0.9755	0.9755	0.9755	0.8452
MDA (nmol/mL)	1.30	0.30	0.39	1.10	<0.0001	<0.0001	0.1817	0.7627	<0.0001	0.0001
Plasma										
GSH-Px (U/mgprot)	1753.52	2039.84	2025.00	1980.00	0.006	0.0088	0.0277	0.9962	0.8181	0.911
CAT (U/mg)	3.26	2.89	3.91	3.97	0.738	0.3429	0.2718	0.0698	0.0523	0.9982
T-SOD (U/mL)	69.42	70.07	70.50	69.39	0.9677	0.8727	>0.9999	0.9899	0.9634	0.8637
T-AOC (mM)	0.84	1.03	1.04	0.76	0.1835	0.1466	0.7653	0.9989	0.036	0.028
MDA (nmol/mL)	3.20	3.94	3.74	3.79	0.6067	0.8027	0.7546	0.986	0.9943	0.9997

BAS: basal diet; Amy: BAS + amylase; Cr: BAS + chromium propionate; Amy-Cr: BAS + amylase + chromium propionate. Differences between treatment groups were determined using Tukey’s multiple comparison test. Statistical significance was set at *P < 0.05*. On day 56, 16 birds per treatment group were collected from the wing vein.

### Serum biochemistry parameters

Plasma biochemical parameters were shown in Table 7. Compared with Group BAS, the HDLC concentrations were increased in Group Amy (*P < 0.0001*), Group Cr (*P = 0.0005*), and Group Amy-Cr (*P = 0.0003*). Meanwhile the concentration of TG was lower in Group Amy (*P = 0.014*), Group Cr (*P = 0.0163*), and Group Amy-Cr (*P = 0.0439*) than in the BAS group. However, there were no significant differences among these groups. The TP levels were increased in Group Cr (*P = 0.0016*) and Group Amy-Cr (*P = 0.0033*) compared with Group BAS. The TP concentration of Group Amy was lower than that Group Cr (*P = 0.0343*). However, it was significantly different from that of Group BAS (*P = 0.3285*). The IgA levels were significantly elevated in Group Amy, Group Cr, and Group Amy-Cr compared with Group BAS (*P < 0.0001*). Additionally, the IgA concentration in Group Amy was higher than that in Group Cr (*P = 0.0338*) and Group Amy-Cr (*P = 0.0106*). The IgG concentrations were significantly increased in Group Amy (*P = 0.0027*), Group Cr (*P = 0.0041*), and Group Amy-Cr (*P = 0.0067*) compared with Group BAS. No significant differences were observed in plasma concentrations of ALT, AST, LDH, ALM, IgM, CRH, CK, or CORT among all experimental groups.

**Table 7:** Effects of dietary amylase, Cr Prop, and their combination on serum biochemical indices in yellow-feathered broilers.

Items	BAS	Amy	Cr	Amy-Cr	*P-*value					
					BAS vs. Amy	BAS vs. Cr	BAS vs. Amy-Cr	Amy vs. Cr	Amy vs. Amy-Cr	Cr vs. Amy-Cr
TCH (mmol/L)	3.55	3.31	3.98	3.76	0.983	0.9066	0.9875	0.7398	0.9009	0.985
HDLC (mmol/L)	4.25	6.86	6.10	6.21	<0.0001	0.0005	0.0003	0.0752	0.1361	0.9732
LDLC (mmol/L)	2.31	1.84	2.32	2.47	0.6942	>0.9999	0.9804	0.6811	0.4867	0.9837
TG (mmol/L)	0.74	0.38	0.39	0.45	0.014	0.0163	0.0439	0.9994	0.8375	0.8866
ALB (g/L)	18.42	20.80	18.02	17.63	0.2491	0.9888	0.9201	0.1461	0.0807	0.9886
TP (g/L)	26.78	29.38	34.03	33.40	0.3285	0.0016	0.0033	0.0343	0.0722	0.9719
ALT (U/L)	1.76	1.17	1.19	1.49	0.0854	0.2237	0.7365	0.8956	0.3435	0.7025
AST (U/L)	12.41	12.86	13.06	12.38	0.8747	0.7125	>0.9999	0.987	0.8556	0.688
Glu (mmol/L)	10.12	10.12	10.85	10.26	>0.9999	0.4208	0.9896	0.4291	0.9911	0.5905
LDH (U/L)	472.51	448.49	448.10	425.59	0.705	0.695	0.2021	>0.9999	0.7336	0.7433
IgA (μg/mL)	167.75	207.52	197.1	194.54	<0.0001	<0.0001	<0.0001	0.0338	0.0106	0.8267
IgM (μg/mL)	200.54	206.25	193.11	192.64	0.9333	0.8679	0.8468	0.559	0.5329	>0.9999
IgG (μg/mL)	418.19	507.86	501.81	495.33	0.0027	0.0041	0.0067	0.9817	0.8686	0.9778
CRH (pmol/L)	43.61	37.53	41.41	40.41	0.2009	0.8528	0.6643	0.529	0.7304	0.9824
CK (U/L)	15.71	13.67	14.22	16.66	0.4043	0.6396	0.8652	0.9685	0.1491	0.2715
CORT (nmol/L)	123.75	134.45	136.66	135.09	0.475	0.3306	0.4294	0.9886	0.9997	0.9959

BAS: basal diet; Amy: BAS + amylase; Cr: BAS + chromium propionate; Amy-Cr: BAS + amylase + chromium propionate. Differences between treatment groups were determined using Tukey’s multiple comparison test. Statistical significance was set at *P < 0.05*. On day 56, liver tissues and serum samples from 8 birds per treatment group were analyzed for antioxidant activity.

### Intestinal morphology

Villus morphology parameters were presented in Table 8. Compared with Group BAS, villus height was significantly increased in the jejunum (*P = 0.0044*) and ileum (*P = 0.0141*) of birds treated with amylase Group Amy. Other parameters showed no differences among treatment groups, regardless of the intestinal segment.

**Table 8:** Effects of dietary amylase, Cr Prop, and their combination on intestinal morphology in yellow-feathered broilers.

Items	BAS	Amy	Cr	Amy-Cr	*P-*value					
					BAS vs. Amy	BAS vs. Cr	BAS vs. Amy-Cr	Amy vs. Cr	Amy vs. Amy-Cr	Cr vs. Amy-Cr
Duodenum										
VH (μm)	1469.71	1610.02	1542.25	1472.74	0.7665	0.9688	>0.9999	0.9743	0.7779	0.9724
CD (μm)	135.44	106.58	134.06	115.65	0.6876	>0.9999	0.8688	0.718	0.9845	0.8908
V:C	10.15	16.15	12.89	14.29	0.1072	0.6682	0.3475	0.5401	0.8622	0.9333
Jejunum										
VH (μm)	1015.72	1259.74	1120.19	1053.12	0.0044	0.2917	0.907	0.1116	0.0141	0.6392
CD (μm)	105.55	91.73	78.53	97.44	0.7571	0.2638	0.9357	0.7809	0.9758	0.5474
V:C	10.00	13.73	14.5	11.82	0.3235	0.1894	0.8174	0.9819	0.7969	0.5882
Ileum										
VH (μm)	926.56	1113.12	965.47	944.38	0.0323	0.9058	0.9894	0.102	0.0551	0.9827
CD (μm)	100.87	86.81	85.98	79.72	0.8247	0.7993	0.5835	>0.9999	0.9715	0.98
V:C	9.52	12.87	12.83	12.23	0.5405	0.5487	0.6911	>0.9999	0.9935	0.9945

BAS: basal diet; Amy: BAS + amylase; Cr: BAS + chromium propionate; Amy-Cr: BAS + amylase + chromium propionate. Differences between treatment groups were determined using Tukey’s multiple comparison test. Statistical significance was set at *P < 0.05*. On day 56, segments of duodenum, jejunum, and ileum from 8 birds per treatment group were collected for histomorphometric analysis.

### mRNA expression of tight junction genes

mRNA expression levels of tight junction genes in the jejunum were presented in Table 9. Compared to the BAS group, the mRNA expression of Occludin was significantly upregulated in Group Amy (*P = 0.0135*), Group Cr (*P < 0.0001*), and Group Amy-Cr (*P = 0.0007*). Notably, the mRNA expression of Occludin was higher in Group Cr than that in Group Amy and Group Amy-Cr. The mRNA expression of Claudin-1 was significantly elevated (*P < 0.0001*) in Group Amy, Group Cr, and Group Amy-Cr compared with Group BAS. The expression of ZO-1 gene in Group Cr was significantly higher than that in Group BAS (*P = 0.0005*), Group Amy (*P = 0.0004*), and Group Amy-Cr (*P = 0.001*). However, the expression of MUC-2 and ZO-2 genes remained unaffected (*P > 0.05*) by treatment with amylase, Cr Prop, or their combination.

**Table 9:** Effects of dietary amylase, Cr Prop, and their combination on mRNA expression of Occludin, Claudin-1, MUC-2, ZO-1 and ZO-2 in the jejunum.

Items	BAS	Amy	Cr	Amy-Cr	*P-*value					
					BAS vs. Amy	BAS vs. Cr	BAS vs. Amy-Cr	Amy vs. Cr	Amy vs. Amy-Cr	Cr vs. Amy-Cr
Occludin	1.00	1.69	2.53	1.95	0.0135	<0.0001	0.0007	0.0024	0.5782	0.0414
Claudin-1	1.00	2.25	2.4	2.12	<0.0001	<0.0001	<0.0001	0.7757	0.8257	0.2961
MUC-2	1.00	1.11	1.24	1.24	0.6133	0.0651	0.0726	0.494	0.526	>0.9999
ZO-1	1.00	1.01	1.47	1.05	0.9997	0.0005	0.9903	0.0004	0.9795	0.001
ZO-2	1.00	1.02	1.08	1.05	0.999	0.9627	0.9919	0.9862	0.999	0.9972

BAS: basal diet; Amy: BAS + amylase; Cr: BAS + chromium propionate; Amy-Cr: BAS + amylase + chromium propionate. Differences between treatment groups were determined using Tukey’s multiple comparison test. Statistical significance was set at *P < 0.05*. Jejunal tissues were collected from 8 birds per treatment group to assess the mRNA expression of tight junction genes.

## Discussion

Starch is the primary energy source in broiler diets, and measures of increasing its degradation will have a beneficial effect on growth performance of broilers. Supplementing with exogenous amylase in broiler diets is an effective method to improve the digestibility of starch. In the present study, amylase supplementation significantly improved various growth performance indexes across different growth phases including ADFI and FCR during the starter period, ADG and BWG during the grower period, and ADG, BWG, and FCR during the finisher period. Overall, amylase supplementation significantly increased ADG, ADFI, and BWG, but had no effect on FCR. Our results are partially consistent with previous studies on amylase supplementation in broilers. Stefanello et al., (2017, 2019) reported that the addition of amylase increased BWG from day 1 to day 40 or from day 14 to day 25, decreased FCR, and did not affect feed intake. Similarly, Aderibigbe et al., (2020) found significant improvements in BWG and feed efficiency in broilers fed amylase-supplementation diets across four post-hatching growth phases. However, these findings contrast with those of Khadija S. Radhi et al., (2023), who found no significant impact on BWG, FCR, or feed intake with amylase supplementation across two growth phases (1–21 days and 22–35 days). Wang et al., (2020) also reported no significant effects of amylase supplementation on BWG, ADG, ADFI, or FCR in yellow-feathered broilers from 21 to 42 days and 43 to 63 days. These findings suggest that although young birds primarily utilize energy from starch-based diets post-hatching, carbohydrate composition is essential factor leading to the variance in energy utilization of feed ingredients by broilers. Besides, the source, environment resistance, type, and dosage of amylase may also have impact to the function in broilers.

The present study confirmed that the addition of Cr Prop in the basal diet could be used to improve growth performance of broiler chickens. Although Brooks et al., (2016) found no effect of Cr Prop supplementation on BWG or feed efficiency, in several studies, Cr Prop supplementation in soybean-maize diets has been shown to improve growth performance of broiler chickens, including increased BWG and reduced FCR (Sahin et al., 2003; Toghyani et al., 2006; V. Van Hoeck et al., 2020). These studies suggest that Cr Prop supplementation could affect various indices of growth performance of broiler under varying rearing conditions. Yellow-feathered broilers have lower growth rates and higher stress levels compared to white broilers, which may facilitate the positive effects of Cr Prop.

One objective of this study was to evaluate the effect of an amylase in combination with Cr Prop on growth performance of broiler chickens fed with soybean-maize based diets from 1 d to 56 d. Unfortunately, no synergistic effects were observed with the combined supplementation of amylase and Cr Prop in the whole stage despite the positive effects of each supplement on broiler growth when administered separately. When examining different stages, we found that the combined use of amylase and Cr Prop significantly decreased ADFI but improved FCR in the starter stage. No other performance differences were observed in the grower and finisher stages. In the starter stage, the body of the bird is small, fast release and absorption of glucose may exceed the utilization capability which may negatively inhibit the feed intake. At the same time, both amylase and Cr Prop can reduce stress of birds, this will give birds better feed conversion ratio.

The present study did not observe any significant effects of either amylase, Cr Prop, or their combination on carcass characteristics in broilers. Our results are in agreement with many researchers who found no significant variation when broilers are fed diets supplementation with amylase (Torres et al., 2003, Fortes et al., 2012, Bedford et al., 2012, Cowieson et al., 2012, and Castro et al. 2019). In contrast, some studies have reported significant positive effects of Cr Prop supplementation on carcass traits (Sahin et al., 2003; Anandhi et al., 2006; V. Van Hoeck et al., 2020). Carcass traits are more related to breeds, feed amino acid or protein levels, growth periods, and body weight. Amylase and Cr Prop mainly facilitate energy metabolism. Therefore, no changes among different treatment groups might be reasonable.

Meat quality is a key concern for both consumers and the poultry industry (Sokolowicz et al., 2016). Drip loss is a critical parameter for evaluating muscle water-holding capacity (Otto et al., 2006). In the present study, drip loss was lowest in the amylase group, followed by the combination group, the basal diet group, and highest in the Cr Prop group. These results suggest that amylase supplementation alone may reduce drip loss rate in the breast muscle of yellow-feathered broilers. However, Khadija S. Radhi et al., (2023) reported no positive effect of amylase on growth performance and meat quality in broilers, which may be attributed to the differences in the enzymes used. Additionally, Cr Prop had no effect on meat quality parameters in this study. This finding contrasts with the results of Xiao et al., (2017) but aligns with those of Huang et al., (2016) in broilers. Notably, in swine, chromium has been reported to reduce drip loss (Matthews et al., 2005; Tian et al., 2015). Since yellow-feathered broilers have a longer growth period and are valued for their meat quality, the minimal changes in meat quality parameters observed on day 56 may be expected. However, if these carcass characteristics were monitored on day 22 or 43, or under stress conditions, the results might differ significantly.

Oxidative stress (OS) has been identified as a crucial factor contributing to declines in growth performance and meat quality in broilers (Xu et al., 2022). OS results from an imbalance between the normal products of aerobic metabolism and antioxidant (Sies, 1997). In this study, we found that amylase supplementation primarily enhanced antioxidant capacity in the breast muscle. This effect may partially account for the observed reduction in drip loss. To our knowledge, no previous studies have investigated the antioxidation properties of amylase in broilers. We suppose that this effect might originate from amylase’s capacity to alleviate the detrimental effects of resistant starch in the digestive tract. Trivalent Cr (Cr^3+^) has strong antioxidant properties and is commonly used in poultry diet (Kroliczewska et al., 2004) to mitigate oxidative stress by enhancing the antioxidant defense system and reducing lipid peroxidation (Preuss et al., 1997). Here we found that dietary Cr Prop supplementation increased GSH-Px, CAT, and T-AOC activity mainly in liver. It’s in agreement with studies of Tawfeek et al., (2014) and Youssef et al., (2022). Zhang et al., (2024) also confirmed that Cr Prop increased T-AOC levels and GSH-Px activity that eliminated peroxides and hydroxyl radicals. Since the liver serves as the target organ for metal element metabolism, chromium tends to accumulate in this organ to exert its antioxidant effects. This explains why chromium supplementation fails to reduce drip loss in breast muscle. Although both amylase and Cr Prop supplementation individually enhanced antioxidant activity, their combination did not yield additional benefits in terms of antioxidant capacity.

Chromium plays a crucial role in carbohydrate metabolism, lipid metabolism, and protein synthesis (Mertz, 1993). This study found that Cr Prop supplementation significantly lowered serum TG but elevated HDLC levels, without affecting total cholesterol or LDLC concentrations. Chromium in organic form has been shown to reduce serum TG and LDLC concentrations, whilst increasing HDLC concentrations in broilers either under heat-stress or normal rearing condition (Kim et al., 1995; Moeini et al., 2011). In partial agreement with previous findings (Lien et al., 1999; Xiao et al., 2016; Zheng et al., 2016). Interestingly, amylase supplementation also induced higher HDLC levels, with higher concentrations of IgA and IgG, compared to Cr Prop alone, but lowered TG level. The digestive tract serves as the largest immune organ in animals. Since energy provision is essential for protein synthesis (including antibody production), amylase supplementation may positively influence immune function by enhancing metabolic energy availability. However, the combined administration of amylase and Cr Prop showed minimal immunomodulatory effects, with only serum IgA levels demonstrating significant improvement.

Previous studies showed that feed efficiency and weight gain were negatively affected by an impairment of the intestinal epithelial barrier (Kogut et al., 2008). In the present study, villus height in both jejunum and ileum were significantly increased in amylase and Cr Prop supplementation groups, with the amylase group showing the greatest villus height. There was little effect of amylase combined with Cr Prop on promoting villi growth. Hayat et al., (2019) investigated the effects of Cr Prop supplementation in broiler diets on intestinal villi growth and found significantly greater crypt depth in jejunum and ileum, as well as significant increase in the thickness of the jejunal wall. However, Makanjuola (2012) and Sandikci et al., (2004) showed that Cr supplementation had no significant impact on crypt depth in broiler diets. Although the findings of this study were not in line with those of previous reports, various studies have demonstrated the positive effects of Cr Prop supplementation on intestinal morphology. The results of intestinal histology slides can be influenced by many factors, including the sampling site, cutting angle, and processing techniques. To further evaluate intestinal health, gene expression analysis was incorporated into this study.

The tight junctions are multi-protein complexes, such as occludin, claudins, and zonula occludens are crucial for the integrity and function of the epithelial barrier. The epithelial barrier system not only links cells but also forms channels which allowing permeability of essential ions, nutrients, and water, on the other hand restricts the entry of bacterial toxins and pathogens (Vicente et al., 2001; Gonzalez-Mariscal et al., 2003). In the present study, the mRNA expression of Occludin and Claudin-1 were significantly enhanced in amylase supplementation, Cr Prop supplementation, and their compound group when compared with the control group. ZO-1 gene expression was upregulated exclusively in the Cr Prop and Amy-Cr supplementation groups. To our knowledge, no published data existed on the effects of amylase and Cr Prop on tight junction gene expression. We hypothesize that these novel findings may partially elucidate their growth-promoting mechanisms.

## Conclusion

In conclusion, amylase supplementation enhanced the growth performance and reduce meat drip loss of yellow-feathered broilers, likely mediated by upregulated antioxidant capacity in the breast muscle and enhanced intestinal tight junction gene expression. In contrast, Cr Prop supplementation improved growth performance primarily through the upregulation of tight genes. However, combined supplementation of amylase and Cr Prop did not exhibit synergistic effects; instead, they appeared to interfere with each other’s efficacy. Further research is needed to elucidate the underlying mechanisms.

## Declaration of competing interests

The authors declare that they have no known competing financial interests or personal relationships that could have appeared to influence the work reported in this paper.

## Appendix Supplementary materials

Image, application 1
