# Supplementary material for: The effect of amylase, chromium propionate and their combination supplementation on growth performance, carcass traits, serum parameters, antioxidant capacity and intestinal health in yellow feathered broilers
Source: Poult Sci. 2025 Apr 29;104(7):105229. doi: 10.1016/j.psj.2025.105229 (PMC12099875; doi:10.1016/j.psj.2025.105229)
Supplement: Supplementary file 1 [file mmc1.pdf]

Researcher Academy researcheracademy.com

ELSEVIER

Certificate of Completion

This certifies that

Kun Chen

has successfully completed the following module

What is plagiarism and how to avoid it (13 minutes)

on Sunday 02 February, 2025

Presented by Tess Bird, PhD

Laura Hassink

Managing Director, Science,
Technology & Medical Journals,
Elsevier
